# Expanded insights into the mechanisms of RNA-binding protein regulation of circRNA generation and function in cancer biology and therapy

**DOI:** 10.1016/j.gendis.2024.101383

**Published:** 2024-08-03

**Authors:** Lixia Li, Chunhui Wei, Yu Xie, Yanyu Su, Caixia Liu, Guiqiang Qiu, Weiliang Liu, Yanmei Liang, Xuanna Zhao, Dan Huang, Dong Wu

**Affiliations:** a Cancer Hospital, Affiliated Hospital of Guangdong Medical University, Zhanjiang, Guangdong 524000, China; b Department of Respiratory and Critical Care Medicine, Affiliated Hospital of Guangdong Medical University, Zhanjiang, Guangdong 524000, China

**Keywords:** Biogenesis, Circular RNAs, Interaction, RNA binding proteins, Tumor microenvironment

## Abstract

RNA-binding proteins (RBPs) regulate the generation of circular RNAs (circRNAs) by participating in the reverse splicing of circRNA and thereby influencing circRNA function in cells and diseases, including cancer. Increasing evidence has demonstrated that the circRNA-RBP network plays a complex and multifaceted role in tumor progression. Thus, a better understanding of this network may provide new insights for the discovery of cancer drugs. In this review, we discuss the characteristics of RBPs and circRNAs and how the circRNA-RBP network regulates tumor cell phenotypes such as proliferation, metastasis, apoptosis, metabolism, immunity, drug resistance, and the tumor environment. Moreover, we investigate the factors that influence circRNA-RBP interactions and the regulation of downstream pathways related to tumor development, such as the tumor microenvironment and N6-methyladenosine modification. Furthermore, we discuss new ideas for targeting circRNA-RBP interactions using various RNA technologies.

## Introduction

RNA-binding proteins (RBPs) are a conserved class of proteins found in eukaryotes.^1^ RBPs form ribonucleoprotein (RNP) complexes through their specific RNA binding domains (RBDs) and play crucial roles in transcriptional and post-transcriptional gene regulation, which functions in key processes such as RNA maturation, turnover, localization, and translation.^2^^,^^3^ Recent studies have shown that some RBPs interact with intronic complementary sequences in precursor messenger RNA (pre-mRNA), affecting the back-splicing of circular RNAs (circRNAs).

circRNAs are a distinct type of RNA and are formed through back-splicing that covalently links the 5′ and 3′ ends of a linear RNA precursor.^4^ circRNAs were previously thought to be a byproduct of splicing errors until the landmark discovery in 2013 of ciRS-7/CDR1as (also known as circRNA sponge for miR-7).^5^ This discovery has made circRNA-microRNA (miRNA) interactions a hot research topic. In addition to functioning as a miRNA sponge, circRNA also exhibits protein sponge, bait, scaffold, and recruiter activity. circRNAs can regulate protein-RNA interactions by binding to specific proteins and isolating them to appropriate subcellular locations. Compared with studying circRNA-miRNA interactions, exploring the regulatory network of circRNA-protein interactions is more complex and interesting. Some proteins play an integral role in the generation of circRNA from biogenesis to degradation. Hence, dysregulation or mutation in RBPs may lead to erroneous binding with circRNAs, thereby altering regulatory pathways and influencing physiological processes and pathological processes of diseases including cancer.^6^^,^^7^

Cancer is a complex and heterogeneous disease. Tumor cells hijack post-transcriptional mechanisms that allow protein expression levels to adjust rapidly and robustly in response to intracellular and extracellular signals, enabling cells to adapt to the local microenvironment. The tumor microenvironment (TME) also influences the growth and evolution of cancer cells.^8^ Tumors and TME constantly interact with each other in a "seed-soil” relationship. Studies have shown that RBPs undergo highly dynamic interactions with circRNAs in the TME.^9^ RBPs interact with circRNAs to modulate downstream gene expression, thereby influencing the expression and function of oncoproteins and tumor suppressor proteins.^10^ Currently, 1542 RBP genes have been identified in the human genome through genome-wide screening, accounting for approximately 7.5% of all protein-coding genes. The number of circRNAs is equally large, suggesting that RBP-mediated biogenesis and functional regulation of circRNAs may be complex. Hence, deciphering the complex network of circRNA-RBP interactions and their cancer-related targets will provide a better understanding of tumor biology and potentially reveal new targets for cancer therapy.

In this review, we discuss the role of RBPs in the generation and functionality of circRNAs. Furthermore, we have summarized the biological significance and regulation of circRNA-RBP interactions and the roles of circRNA-RBP in tumorigenesis, along with the factors influencing their interactions and functions. Moreover, we have explored the potential of RBPs and circRNAs as targets for therapeutic interventions in cancer. Finally, we discuss potential strategies for targeting RBPs and circRNAs for cancer treatment and the possible clinical applications.

## The crosstalk between circRNAs and RBPs

### Biogenetic patterns of circRNA

circRNAs can be classified into three categories based on the products of pre-mRNA splicing. The first category comprises exonic circRNAs; these are predominantly localized in the cytoplasm and arise from the reverse splicing of exonic regions within pre-mRNA. The second category encompasses intronic circRNAs; these are primarily located in the nucleus and formed via lariat-driven circularization. The third category includes exon-intron circRNAs; these are also mainly found in the nucleus and generated from both exonic and intronic elements.^11^, ^12^, ^13^

There are three proposed models for the formation of circRNAs: i) intron pairing, where pre-mRNA forms a circular structure by covalently linking the downstream 5′ splice site of an exon with the upstream 3′ splice site, followed by the removal of the intron through splicing, resulting in a closed-loop circRNA; ii) lariat-driven circularization, which occurs when the removed introns during pre-mRNA splicing form lariats; and iii) RBP-driven circularization, where the biogenesis of circRNAs is influenced by trans-acting factors such as RBPs, which bind to intronic complementary sequences or other sequences within the pre-mRNA to facilitate circRNA production.^14^, ^15^, ^16^ Several RBPs, including muscleblind, quaking (QKI), adenosine deaminase acting on RNA (ADAR), fused in sarcoma (FUS), heterogeneous nuclear ribonucleoproteins (hnRNPs), and serine–arginine rich proteins, have been shown to regulate the biogenesis of circRNAs.^13^

### Structural features of RBPs involved in circRNA-RBP interactions

RBPs recognize and bind to specific consensus motifs or structural elements within RNA molecules through a unique modular arrangement of RBDs.^1^ These well-defined RBDs include RNA recognition motifs, K-homologous (KH) domains, and DEAD-box RNA helicase domains.^1^

**Figure 1:**
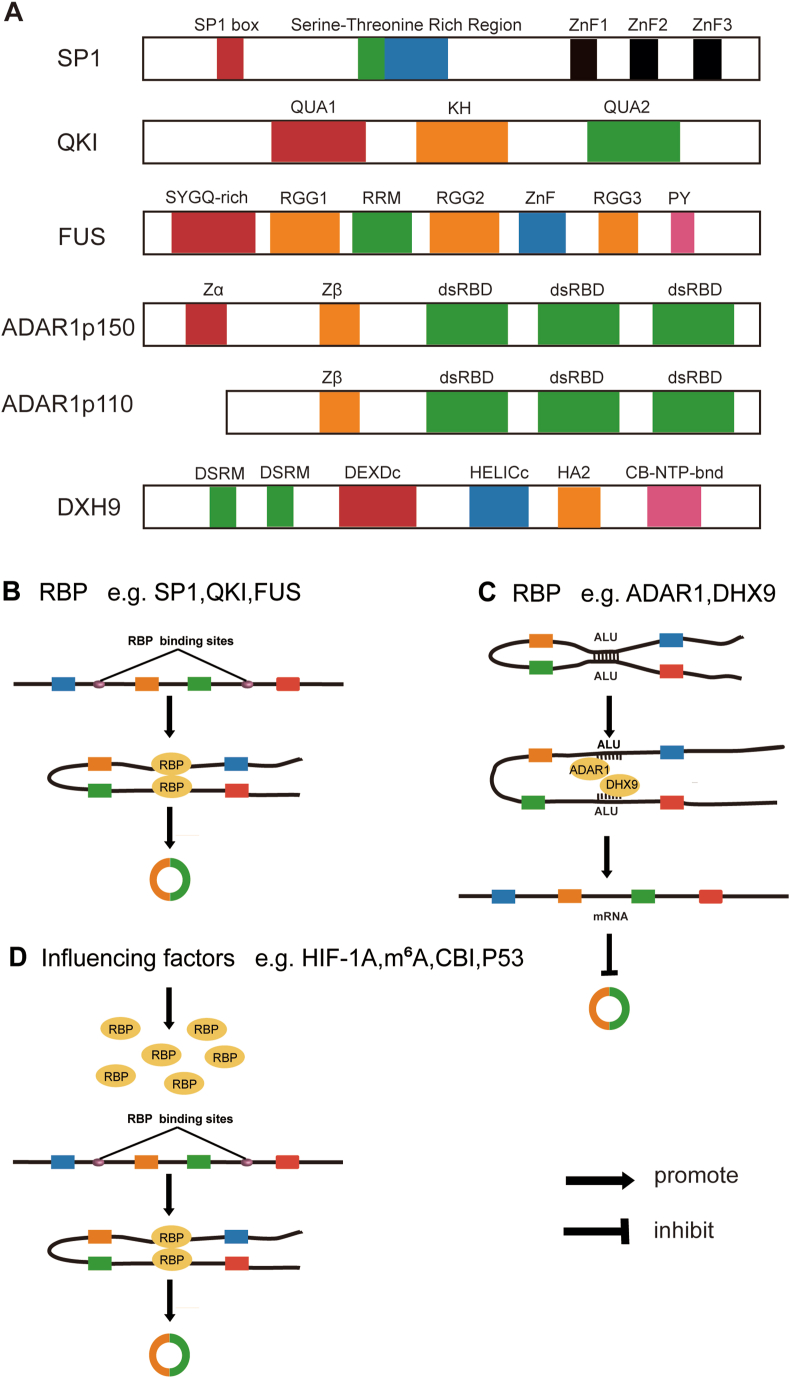
The interaction between circular RNA (circRNA) and RNA-binding protein (RBP). **(A)** RBP binds to circRNA by RNA binding domain (RBD). The common RBDs include zinc finger (ZNF) domains, K-homologous (KH) domain, RNA recognition motif (RRM), double stranded RNA binding domain (dsRBD), and DEAD-box RNA helicase domains. Each RBD is drawn as colored boxes. **(B)** The way RBP promotes circRNA genesis. The dimerization of RBP is beneficial to connect the upstream and downstream splicing donor sites and promote the reverse splicing of circRNA (*e.g.*, FUS (fused in sarcoma), QKI (quaking), and SP1 (specificity protein 1)). **(C)** The way RBP inhibits circRNA genesis. RBPs such as ADAR1 (adenosine deaminase acting on RNA 1) and DHX9 (DExH-box helicase 9) disrupt Alu repetitive sequences, allowing the splicing machinery to produce linear mRNA. **(D)** The factors affecting the binding of RBPs to circRNAs. Relevant factors in tumors can promote the binding of RBPs to circRNAs and facilitate the reverse splicing of circRNA, such as HIF1α (hypoxia-inducible factor 1 subunit alpha), m^6^A (N6-methyladenosine), CB1 (cannabinoid type I receptor), and P53.

Nearly half of all RBPs bind to RNA in a sequence- and structure-specific manner.^7^ For instance, QKI and hnRNPs interact with RNA through their KH RBDs, whereas adenosine deaminase acting on RNA 1 (ADAR1) harbors a Z-DNA binding domain at its N-terminus, employing a left-handed conformation for DNA/RNA binding.^17^ The other half of RBPs have been found to lack canonical RBDs, and each RBP engages with RNA in a unique manner.^1^ In this review, we focus on the RBPs with classical RBDs in the circRNA-RBP network that play key roles in tumor biology (Fig. 1). A list of circRNA-RBP and their biological functions in different cancers types is reported in Table 1.

**Table 1:** The list of circRNA-RBP and their biological functions in different cancers types.

RBP	CircRNA biogenesis	CircRNA	Cancer type	Biological function	Mechanism	Refs
QKI	promote	circNOTCH128	Non small cell lung cancer	proliferation↑	GPER regulated circNOTCH1 expression via transcriptional regulation on QKI by YAP1/TEAD complex.	28
SP1,FUS	promote	circ_0026628	colorectal cancer	cell roliferation, migration, EMT, and stemness↑	Circ_0026628 activate the Wnt/β-catenin/SOX2 pathway via sponging miR-346 and recruiting FUS, SOX2 activated SP1 to induce circ_0026628 upregulation.	32
SP1	promote	circSCAF11	glioma	angiogenesis↑	The positive regulation of circSCAF11/miR-421/SP1/ VEGFA.	33
SP1	promote	circ_0005529	gastric cancer	growth and metastasis ↑	The signal axis of circ_0005529/miR-527/Sp1	34
SP1	promote	circZNF609	nasopharyngeal carcinoma	growth and metastasis↑	Circ-ZNF609 absorbing mir-150-5p and upregulating SP1.	35
SP1	promote	circSNX25	triple-negative breast cancer	cell proliferation↑	SP1 enhance circSNX25 biogenesis	37
SP1	inhibit	circ_0001875	non-small cell lung cancer	cell roliferation, migration, EMT↑	SP1 negatively regulated circ-0001875 formation, which was disrupted by competitive binding of HIF1α to SP1 under hypoxia condition.	38
FUS	promote	circROBO1	breast cancer	metastasis ↑	circROBO1/KLF5/FUS positive feedback loop.	45
FUS	promote	circ_002136	gliomas	angiogenesis ↑	FUS/circ_002136/miR-138-5p/SOX13 positive feedback loop.	46
FUS	promote	circAAGAB	breast cancer	migration and invasion↓	The stability of circAAGAB increased by binding with FUS.	47
ADAR1	inhibit	circNEIL3	pancreatic cancer	cell proliferation and metastasis↑	The expression of circNEIL3 is regulated by ADAR1 through a circNEIL3/miR-432-5p/ADAR1/ GLI1 axis. negative feedback loop.	54
ADAR1	inhibit	circ_0004872	gastric cancer	cell roliferation, invasion, and migration↓	Forming a regulatory feedback loop of Smad4/ADAR1/ hsa_circ_0004872/miR-224/ Smad4.	48
DHX9	inhibit	circDCUN1D4	lung cancer	invasion and metastasis↓	CircDCUN1D4/HuR/TXNIP form an RNA-protein ternary complex, DHX9 suppress the biogenesis of circDCUN1D4.	61
DHX9	inhibit	circCCDC66	colorectal cancer	cell roliferation, invasion and migration↑	The expression of circCCDC66 is induced by oxaliplatin through PI3KK-mediated DHX9 phosphorylation.	63
FUS	promote	circTBC1D14	triple-negative breast cancer	cell roliferation, migration, and invasion↓	FUS interacts with circTBC1D14 to induce cyclization, hypoxic conditions can induce FUS-circTBC1D14-associated SG formation after modification by protein PRMT1.	71
HSP90	promote	CircSHKBP1	gastric cancer	cell roliferation, migration, invasion and angiogenesis↑	CircSHKBP1 directly bound to HSP90 and obstructed the interaction of STUB1 with HSP90, inhibiting the ubiquitination of HSP90.	72
KHSRP	promote	circLMP2A	Epstein-Barr virus associated gastric cancer	cell invasion, metastasis, angiogenesis↑	Ebv-circLMP2A interacted with KHSRP to enhance KHSRP-mediated decay of VHL mRNA, leading to the accumulation of HIF1α under hypoxia.	73
QKI, IGF2BP	inhibit	circNDUFB2	non-small cell lung cancer	anti-tumor immunity ↑	Decrease of QKI contributes to the downregulation of circNDUFB2, TRIM25/circNDUFB2/IGF2BPs form ternary complex facilitates ubiquitination and degradation of IGF2BPs.	77
PCBP2, YTHDC1	promote	circCPSF6	hepatocellular carcinoma	cell invasion, metastasis, proliferation↑	circCPSF6 facilitated YAP1 expression by binding to PCBP2, METTL3/YTHDC1-mediated m6 A modification is involved in the biogenesis of circCPSF6s.	78
METTL3	promote	circCUX1	hypopharyngeal squamous cell carcinoma	release of inflammatory factors ↓	METTL3-mediated the m^6^A methylation of circCUX1 and stabilized its expression.	79
FUS	promote	circCNOT6L	embryo development	spermatogenesis and sperm maturation↑	FUS interaction with CNOT6L and form FUS-QKI-RNApol2 complex, CB1 stimulation increased circCNOT6L.	84
HUR	promote	circ_0006240	lung cancer	EMT↑	In the presence of p53, circ_0006240 interacts with HUR to prevent PTBP1 nuclear export.	85
SRSF1	promote	circCDR1as	lung cancer	cell proliferation, EMT,angiogenesis↑	CircCDR1as bound to SRSF1 and affected the splicing of VEGFA by SRSF1.	86

## circRNA-RBP in cancer

### RBPs promote circRNA biogenesis in cancer biology

#### QKI

QKI, an RBP, is a member of the STAR family.^18^ QKI promotes the biogenesis of circRNAs. QKI contains a STAR domain that consists of a single KH domain flanked by two conserved Qua 1 and Qua 2 domains.^19^ Qua1 is critical for homodimerization and recognizes two RNA regulatory elements simultaneously.^20^ The KH domain cooperates with Qua2 in the interaction with RNA targets, playing a crucial role in RNA binding.^20^^,^^21^ Through its STAR domain, QKI participates in various aspects of RNA homeostasis, including RNA stability, splicing, translation, microRNA (miRNA) processing, and circRNA biogenesis. QKI has been linked to diseases such as breast cancer, lung cancer, and bladder cancer.^19^^,^^22^, ^23^, ^24^

In cancer, tumorigenesis, invasion, metastasis, and drug resistance are often associated with epithelial–mesenchymal transition.^25^ During the epithelial–mesenchymal transition, hundreds of circRNAs change expression, and over one-third of circRNAs are regulated by QKI.^26^ A large number of circRNAs contain QKI binding sites. QKI dimerizes through its N-terminal Qua 1 domain, enabling it to bind to two separate nucleotide sequences located on the same or independent RNA molecules. When QKI binds to recognition elements within introns near circRNA splicing sites, it brings exons into proximity, promoting pre-mRNA splicing and enhancing circRNA biogenesis.^26^

In the TME, QKI not only directly binds to introns of circRNAs to promote circRNA production, but it also indirectly regulates the production of circRNAs by its up-regulation by specific transcription factor complexes or receptors. Yin-Yang 1 (YY1), a transcription factor linked to cancer progression, exhibits a positive correlation with QKI expression in liver cancer. YY1 forms the YY1/p65/p300 complex and binds to enhancers and the promoter of the QKI gene to activate QKI gene transcription.^27^ Up-regulated QKI promotes the biogenesis of related circular RNAs, thus leading to the malignant progression of liver cancer.^27^ Research has shown that YAP1 (Yes-associated protein 1)-TEAD (transcriptional enhanced associate domain) participates in circRNA biogenesis by regulating the transcription of QKI. G protein-coupled estrogen receptor (GPER), also known as G protein receptor, prevents YAP1 phosphorylation to promote YAP1-TEAD action, thereby promoting the generation of circNOTCH1.^28^ GPER is a mechanical modulator of the intercellular matrix that can induce various changes in the TME.^29^ Its potential as an effective mechanical modulator in the TME is emphasized by its targeting of key molecules involved in cellular mechanics, such as Ras homolog gene family, member A (RhoA), myosin regulatory light chain 2 (MCL-2), and YAP, which regulate tumor behavior, including growth, metastasis, and chemotherapy resistance.^29^

#### SP1

Specificity protein 1 (SP1) is one of the most well-characterized transcription factors and contains three highly conserved C2H2-type zinc finger (ZNF) domains. Through its ZNF domains, SP1 recognizes and directly binds to GC-box promoter elements in DNA, thereby promoting gene transcription.^30^^,^^31^ SP1 is often overexpressed in many human cancers, and its expression levels are associated with tumor stage and poor prognosis. Inhibition or knockdown of SP1 expression levels has been shown to reduce tumor formation, growth, and metastasis.^30^

Previous studies have shown that SP1, as a target gene of circRNA, regulates gene transcription after indirect binding to circRNAs, including circZNF609 and circRNA_0005529, circSCAF11, and circRNA_0026,628, to promote cancer occurrence.^32^, ^33^, ^34^, ^35^ Song et al revealed that in addition to DNA binding ability, SP1 also directly binds to RNA *in vivo*.^36^ Thus, SP1 is not only a DNA-binding transcription factor but also an RBP. The authors further demonstrated that SP1 is a regulator of alternative polyadenylation (APA) and exhibits oncogenic potential in breast cancer.^36^ SP1 promotes cell proliferation by shortening the 3′UTR through APA, which is a common phenomenon in tumor cells.^36^ *In vivo*, SP1 binds the 3′UTR of RNAs via its C2H2-ZNF domains, affecting the APA profile of numerous genes, suggesting that SP1 as an RBP may play a role in regulating tumor characteristics.^36^ Furthermore, Gao et al demonstrated that SP1 interacts with circSNX25, enhancing circSNX25 biogenesis and significantly promoting the growth and proliferation of triple-negative breast cancer cells.^37^

Our previous study demonstrated TME's role on SP1 in regulating circRNA biogenesis. Under normoxic conditions, SP1 directly binds to the Alu sequence in the pre-mRNA of FAM120A, reducing the circularization of circ_0001875. However, under hypoxic conditions, the expression of hypoxia-inducible factor 1 subunit alpha (HIF1α) increases, resulting in its competitive binding to the Alu sequence of SP1. This relieves the inhibitory effect of SP1 on circ_0001875 circularization and promotes circ_0001875 generation.^38^ These results provide new insights into the regulatory patterns of circRNA formation by SP1 under hypoxic conditions.

Moreover, circRNAs in the TME also reciprocally regulate SP1 through functioning as a competitive endogenous RNA.^32^ These circRNAs and Sp1 competitively bind to miRNAs and participate in post-transcriptional regulation of protein expression, thus miRNAs act as a bridge in the functional crosstalk between Sp1 mRNAs and circRNAs.^39^ In pancreatic cancer, circ_0026,628 competes with SP1 for binding to miR-346, recruiting FUS to enhance SP1 expression at the post-transcriptional level, thereby strengthening the interaction between SP1 and β-catenin and activating the Wnt/β-catenin pathway. Downstream targets of the Wnt/β-catenin pathway, such as SRY-box transcription factor 2 (SOX2), transcriptionally activate SP1, thereby elevating the levels of circ_0026,628 and promoting pancreatic cancer development.^32^

#### FUS

The FUS gene, located on chromosome 16 in humans, has been identified as a fusion oncogene in human liposarcoma. FUS contains an N-terminal glutamine/glycine/serine/tyrosine-rich domain, a glycine-rich region, an RNA-recognition motif, two arginine-glycine-glycine-rich regions, a ZNF domain, and a C-terminal proline–tyrosine nuclear localization signal.^40^ FUS functions as a DNA/RNA binding protein and is involved in the regulation of RNA metabolism, including in RNA transcription, pre-mRNA splicing, and mRNA transport and translation.^41^^,^^42^

Under normal physiological conditions, FUS is predominantly localized in the nucleus. However, under pathological conditions, FUS mutations occur, causing mislocalization and abnormal cytoplasmic aggregation of FUS, leading to RNA metabolism dysfunction.^41^, ^42^, ^43^ Recent findings have demonstrated that FUS is involved in circRNA synthesis.^43^^,^^44^ FUS regulates changes in circRNA abundance primarily at the post-transcriptional level rather than during transcription.^43^ Lorenzo et al^43^ identified 19 circRNAs expressed in mouse motor neurons derived from *in vitro* sources and determined that the production of these circRNAs is regulated by FUS. FUS preferentially binds to the 5′ ends of extremely long introns and mainly accumulates near exons in genes associated with neuronal function and neurodegeneration. FUS regulates circRNA biogenesis by connecting flanking introns through reverse splicing. This regulation may be associated with alterations in nuclear levels of FUS.^43^

The interaction between FUS and circRNAs leads to a positive feedback loop.^43^ circROBO1 up-regulates Krüppel-like factor 5 (KLF5) by sponging miR-217–5p, enabling the interaction between KLF5 and FUS promoters to activate FUS transcription. This, in turn, promotes the reverse splicing of circROBO1 pre-mRNA, leading to the up-regulation of circROBO1 expression. This circROBO1/KLF5/FUS positive feedback loop accelerates liver metastasis in breast cancer.^45^ Studies on circ_002136 have also confirmed a positive feedback regulatory mechanism of FUS-mediated circRNA generation. FUS expression is positively correlated with SOX13, which promotes FUS transcription by binding to its promoter, thereby mediating the generation of circ_002136.^46^ The positive feedback loop FUS/circ_002136/miR-138–5p/SOX13 regulates angiogenesis in gliomas.^46^ The regulation of circRNA biogenesis by FUS and the crucial role in the progression of various diseases is currently an important research topic in the field of biomedicine.^46^

During the progression of solid tumors, the rapid proliferation of cancer cells exceeds the growth of surrounding blood vessels, resulting in inadequate blood supply and a hypoxic microenvironment. This hypoxic microenvironment can affect the binding between FUS and circRNAs. In breast cancer, FUS binds to circAAGAB under hypoxic conditions, increasing the stability of circAAGAB and enhancing the radiation sensitivity of breast cancer cells through the p38/MAPK pathway.^47^ Elevated levels of FUS exhibit toxic gain-of-function properties by disrupting the homeostasis of proteins and RNA, thereby participating in tumor development and altering the TME.

### RBPs inhibit circRNA biogenesis in cancer biology

#### ADAR1

In recent years, a growing body of evidence has revealed the pivotal role of RNA editing enzymes in the generation and regulation of circRNAs.^48^^,^^49^ ADAR1 is an RNA editing enzyme that catalyzes adenosine-to-inosine (A-to-I) editing on double-stranded RNA (dsRNA). A-to-I editing is an important post-transcriptional modification, and its dysregulation leads to abnormal editing of proteins, which may affect phenotypic changes in cancer.^50^^,^^51^

ADAR1 inhibits circRNA biogenesis through A-to-I editing.^52^ Approximately 88% of circRNAs in the human genome contain Alu repetitive sequences in their flanking introns, which are susceptible to frequent ADAR1-mediated A-to-I editing events. This results in inosine being recognized as guanosine and subsequently forming base pairs with cytidine. This alteration leads to changes in synonymous codons and selective splicing, which ultimately suppresses circRNA circularization.^51^, ^52^, ^53^ ADAR1 also influences circRNA generation by interacting with Alu sequences. Shen et al showed that circNEIL3 regulates ADAR1 to promote proliferation and metastasis of pancreatic cancer cells, thereby playing a crucial role in the progression and prognosis of pancreatic cancer. circNEIL3 expression is negatively regulated by ADAR1. ADAR1 binds to the Alu sequence on the NEIL3 (Nei endonuclease VIII-like 3) pre-mRNA, hindering circNEIL3 biogenesis.^54^ Notably, ADAR1-regulated circRNAs are not just byproducts of reverse splicing; they play a crucial role in circRNA generation, which, in turn, affects oncogenesis and cancer progression.^51^

In the TME, several transcription factors have been found to modulate ADAR1 expression levels. Androgen receptor, a transcriptional activator of the ADAR1 promoter, up-regulates ADAR1 expression, leading to the inhibition of circRNA generation and thereby promoting the development of liver cancer.^55^ Another study on circ_0004872 revealed that the transcription factor Smad 4 directly binds to the ADAR1 promoter region, subsequently reducing ADAR1 expression levels. Smad4 thus impacts the generation of circ_0004872, thereby suppressing gastric cancer cell proliferation, invasion, and migration.^48^

Aberrant A-to-I editing, which results in an imbalanced or dysregulated frequency and efficacy of editing across different genes or transcripts, is referred to as A-to-I imbalanced editing. This A-to-I imbalanced editing can be attributed to mutations of the ADAR gene, changes in the regulation of transcription factors, alterations in the cellular milieu, or other yet unknown factors. This imbalanced editing can lead to alterations in RNA functionality and stability, exerting profound repercussions on the biological processes within cells and the progression of diseases, and has been identified as a tumor promoter across multiple tissue types.^48^

#### DHX9

DExH-box helicase 9 (DHX9), also known as RNA helicase A, is located on chromosome 1.^56^ DHX9 contains a dsRNA binding domain, a helicase conserved C-terminal domain, and an oligonucleotide/oligosaccharide-binding fold nucleotide-binding domain.^57^ DHX9 is a nucleoside triphosphate-dependent unwinding enzyme that acts on DNA and RNA duplexes as well as other complex multi-nucleic acid structures.^58^

DHX9 interacts with the inverted repeat Alu sequences located within intronic regions, thereby resolving inverted repeat Alu-mediated structures and reducing the generation of circRNAs.^59^^,^^60^ Inhibition of DHX9 has been shown to increase the expression of circDCUN1D4 and promote the formation of circDCUN1D4/HuR (Hu-antigen *r*)/TXNIP (thioredoxin interacting protein) ternary complexes. This is beneficial for cirDCUN1D4 to inhibit the metastasis of lung cancer cells by stabilizing thioredoxin-interacting protein levels.^61^ Additionally, studies have suggested a functional connection between ADAR and DHX9; the combined loss of these two enzymes leads to defects in dsRNA accumulation and an increase in circRNA production.^62^

During tumor treatment, DHX9 expression changes may serve as a stress response. In chemoresistant colorectal cancer cells, DHX9 phosphorylation induced by oxaliplatin regulates the elevation of circCCDC66 levels.^63^ Oxaliplatin, a third-generation platinum-based anti-cancer drug, exerts cytotoxicity by inducing DNA damage through multiple pathways.^64^ Upon DNA damage, DHX9 is phosphorylated by and associates with one of the PI3KK (phosphatidylinositol 3-kinase-related kinase) DNA-dependent protein kinases.^63^ The DNA damage response is a fundamental physiological mechanism and activates ataxia telangiectasia and Rad3-related (ATR) and ataxia telangiectasia mutated (ATM) protein kinases.^65^ DHX9 is an ATM/ATR substrate and may be functionally regulated by PI3KK-mediated phosphorylation. It initiates multiple repair actions through cascading reactions.^62^^,^^64^ Damaged DHX9 can promote the expression of oncogenic circRNAs, which help tumor cells escape from the genotoxic stress response. This provides a novel possibility for the regulation of oncogenic circRNA expression through intervention of RBP expression *in vitro*.^63^

The expression levels of DHX9 in the TME are influenced by various factors. For example, membrane-associated ring–CH–type finger 6 (MARCH6) destabilizes DHX9 and activates the AKT (protein kinase B)/mTOR (mammalian target of rapamycin) signaling pathway in thyroid cancer.^66^ In prostate cancer, DHX9 is a transcriptional target of the SOX4 transcription factor. Upon induction by the Wnt signal, SOX4 forms a nuclear complex with plakoglobin, blocking the SOX4-DHX9 interaction and thereby inhibiting the function of DHX9.^67^ The molecular mechanisms that influence DHX9 expression levels may also lead to changes in circRNA expression levels.

### The factors affecting RBP-circRNA interactions in cancer

#### Tumor microenvironment

Cancer development and progression occur in concert with alterations in the local TME.^68^ Cancer cells shape the function of their microenvironment by secreting cytokines, chemokines, and other factors.^68^ RBPs selectively mediate the differential expression of circRNAs in tumors through various mechanisms and reprogram the TME to support tumor survival.^69^

Hypoxia is a crucial factor in the TME and promotes malignancy.^70^ Under hypoxic conditions, RBPs exhibit temporal and spatial specific expression, which can lead to differential expression of circRNAs in tumor tissues.^13^ The hypoxic TME affects the function and expression of RBPs, resulting in significant differences in the expression level of circRNAs between normoxic and hypoxic conditions. circ-0001875 and circAAGAB, described above, are typical examples of circRNAs with altered expression under hypoxic conditions.^38^^,^^47^ Furthermore, hypoxia can impact the binding of RBPs to circRNAs. Liu et al identified a hypoxia-induced circRNA, circTBC1D14, in triple-negative breast cancer. Under hypoxic conditions, circTBC1D14 interacts specifically with protein arginine methyltransferase 1 (PRMT1), forming FUS-circTBC1D14 stress granules by binding to PRMT1-methylated FUS. This interaction further enhances the expression of circTBC1D14. The granules are exported into the cytoplasm, inducing autophagy by recruiting lysosome-associated membrane protein 1 (LAMP1).^71^ Hypoxia can modify the expression or activity of RBPs, exerting specific effects in tumors by impacting the production and functionality of circRNAs. Furthermore, RBPs can be regulated at the transcriptional or post-translational levels, thereby affecting their availability and affinity for binding to circRNAs.

Extensive research has been conducted on differentially expressed circRNAs in the TME. In addition to being regulated by RBPs, circRNAs can modulate the expression of RBPs, resulting in negative feedback mechanisms. Tumors are characterized by their highly proliferative nature, necessitating the rapid establishment of a neovascular network to acquire essential nutrients for growth. Aberrant development of this neovascular network exacerbates tumor hypoxia and increases the risk of metastasis and dissemination. circSHKBP1 is highly expressed in the extracellular vesicles of gastric cancer cells. circSHKBP1 acts as a sponge for miR-582–3p, leading to increased expression of the RBP family member HUR. HUR binds directly to vascular endothelial growth factor (VEGF) mRNA, enhancing the stability of circSHKBP1, and consequently promoting angiogenesis.^72^ Hypoxia is one of the primary factors driving tumor-induced angiogenesis. In Epstein–Barr virus (EBV)-associated gastric cancer, the interaction between KH-type splicing regulatory protein (KHSRP) and the EBV-encoded circLMP2A enhances KHSRP-mediated degradation of von Hippel–Lindau mRNA, resulting in the accumulation of HIF1α under hypoxic conditions. HIF1α promotes angiogenesis by up-regulating VEGFA expression.^73^ This RBP-circRNA pattern plays a pivotal role in regulating angiogenesis in EBV-associated gastric cancer, with the TME playing a crucial role throughout the entire process.

#### N6-methyladenosine (m^6^A)

m^6^A modification is the most common RNA modification in eukaryotes. It plays a crucial role in regulating RNA stability, splicing, and translation.^74^ Dysregulation of m^6^A regulatory factors can lead to imbalanced m^6^A levels in cancer cells. This can result in the dysregulation of oncogenes and tumor suppressor genes, thus impacting cancer development.^75^

Numerous studies have elucidated how m^6^A modification affects the interaction between circRNAs and RBPs, thus influencing tumorigenesis and progression.^73^^,^^76^ For example, in non-small cell lung cancer, circNDUFB2 acts as a scaffold, interacting with the tripartite motif-containing 25 (TRIM25) and insulin-like growth factor 2 mRNA-binding protein (IGF2BP) to form a ternary complex that promotes IGF2BP ubiquitination and degradation; this process is enhanced by m^6^A modification of circNDUFB2.^77^ circNDUFB2 is also recognized by retinoic acid-inducible gene I (RIG-I) to activate RIG-I–MAVS (mitochondrial antiviral-signaling) signaling cascades and recruit immune cells into the TME, participating in the degradation of IGF2BP and activation of anti-tumor immunity during non-small cell lung cancer progression.^77^

The m^6^A modification not only affects the binding of RBPs to circRNAs but also regulates RNA expression. Common RBPs modified by m^6^A are hnRNPs, YTH m^6^A RNA binding proteins (YTHDFs) (including YTHDC1/2 and YTHDF1/2/3), IGF2BPs (such as IGF2BP1/2/3), and zinc finger CCCH-type containing 13 (ZC3H13). m^6^A can facilitate the translation of endogenous circRNAs with the assistance of eukaryotic translation initiation factor 4 gamma 2 (eIF4G2) and YTHDF3.^78^ m^6^A-modified circCPSF6 activates YAP1, driving malignant tumor development in hepatocellular carcinoma, while m^6^A-regulated cirCUX1 binds to caspase 1, suppressing its expression, resulting in reduced release of inflammatory factors and leading to radioresistance in hypopharyngeal squamous cell carcinoma.^78^^,^^79^ Reader proteins bind to m^6^A sites on target RNAs and mediate their modification, thereby regulating RNA expression.^80^, ^81^, ^82^, ^83^

### Relevant factors in cancer

In addition to the extensively studied interplay among circRNA, m^6^A modification, and TME, the interaction between circRNAs and RBPs is modulated by various proteins and specific factors. For example, in the absence of cannabinoid type I receptor (CB1), circCNOT6L and FUS are expressed at low levels in human spermatozoa.^84^ However, upon stimulation of CB1, the production of circCNOT6L increases.^84^ This is attributed to facilitation of the interaction between FUS and CNOT6L mRNA and the formation of FUS-QKI-RNApol2 heterotypic trimeric complexes.^84^ Recent research has revealed that lung cancer cells with wild-type p53 may up-regulate the expression of circ_0006240 after radiation to form a circ_0006240/HUR/PTBP1 (polypyrimidine tract binding protein 1) complex leading to radiotherapy resistance.^85^ In PM2.5-induced lung cancer, circCDR1as specifically binds to splicing factor 1 rich in serine/arginine (SRSF1), affecting SRSF1's splicing of VEGFA mRNA and ultimately inhibiting lung cancer cell apoptosis, thereby promoting the development of PM2.5-induced lung cancer.^86^ Moreover, circ-ZNF609 and circRNAs containing m^6^A modification sites can be translationally induced during heat shock. Additionally, the stability of circMBL-derived peptides increases under conditions of starvation.^87^^,^^88^

### Cancer therapy targeting circRNA-RBP

**Figure 2:**
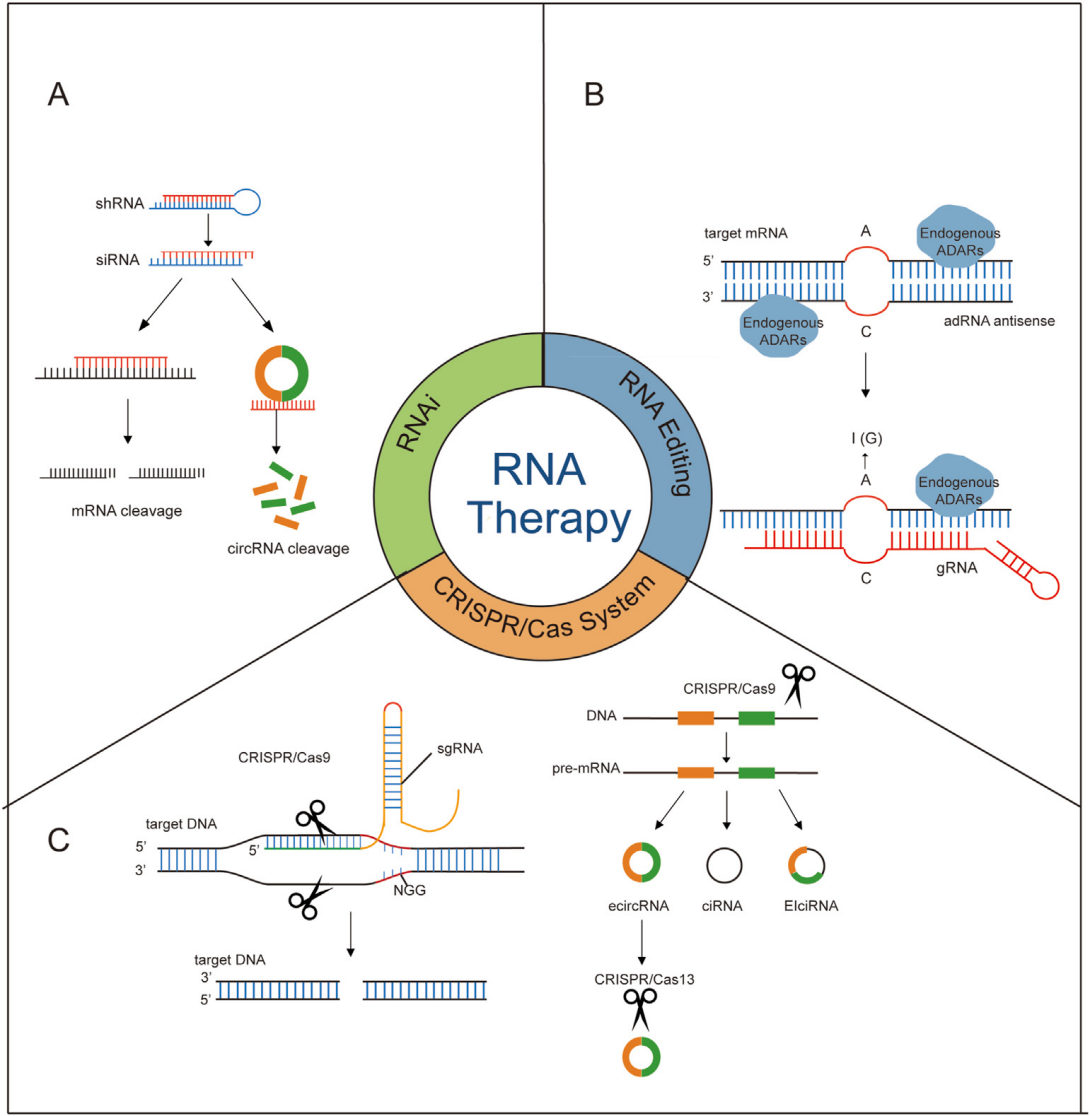
The approaches to RNA therapy targeting the circular RNA (circRNA)-RBP (RNA-binding protein) interactions. **(A)** RNAi: siRNA/shRNA mediate the cleavage of mRNA and circRNA. siRNA/shRNA targeting the back–splice junction of circRNAs induces circRNA cleavage. **(B)** RNA editing: gRNA is used to guide ADAR positioning editing. **(C)** CRISPR/Cas system: CRISPR/Cas9 knocks out circRNA by disrupting intron pairing on both sides of the circular exon, while CRISPR/Cas13 can directly target the back-splicing junction of circRNAs.

The unique domains of RBPs and the stable expression of circRNAs have endowed them with unique characteristics that can be useful for biotechnological applications.^89^ RNA therapy has entered a new era of rapid development with major innovations in biotechnology, such as the rapid development and deployment of mRNA vaccines to combat the COVID-19 pandemic.^90^ Researchers have explored targeting circRNAs and RBPs and attempts to develop RNA therapy that combine the benefits of both. Thus far, a variety of targeted therapies for circRNAs and RBPs have been developed.^91^, ^92^, ^93^ Here we discuss three distinct approaches to RNA-based therapy targeting the circRNA-RBP interactions: RNAi, RNA editing, and the CRISPR/Cas system^6^^,^^8^ (Fig. 2). A list of RNA thrapy targeting circRNA-RBP system is reported in Table 2.

**Table 2:** RNA thrapy targeting circRNA-RBP system.

RNA thrapy	RNA tool	Target gene	Methods	Applications	Refs
RNAi	siRNA , shRNA	circRNA	target circRNA-specific BSJ structure	Knockdown circRNAs *in vivo*.	91, 97
		RBP	control post-transcriptional gene expression through homology-dependent degradation of target mRNA by siRNA	Targeting nanoparticle delivery of HUR-RNAi to inhibit cancer development	98,99
RNA editing	ADARs;	RBP	gRNA for site-specific editing of mRNA	Treating A1-antitrypsin deficiency caused by SERPINA1 missense mutations.	90,100 103,104
		circRNA	circular ADAR-recruiting guide RNA	To improve RNA editing efficiency and extend the editing time.	90
CRISPR-Cas system	CRISPR/ Cas9	circRNA	disrupting intron pairing on both sides of the circular exon	Knocking out circHIPK3 *in vitro*.	107,108
	CRISPR/ Cas13	circRNAs	target circRNA-specific BSJ structure	Knock down the circRNA	89
	CRISPR/ Cas9	RBP	identify RBP molecules by high-throughput CRISPR screening	HNRNPL, METTL3, RBP network in AML	92,110,111

#### RNAi

RNAi technology is currently the most feasible and widely used method among all discussed methods.^91^^,^^94^ RNAi has great potential to target any cancer protein, opening the door to countless possibilities for effective treatment, and holds great promise in the field of cancer treatment.^95^ The development of potential RNAi strategies involves the selection of circRNA, RBP, or both; tumors can be screened for carcinogenic and highly expressed molecules and they can be inhibited by RNAi-mediated silencing.^91^ Short interfering RNA (siRNA) and short hairpin RNA (shRNA) are currently used to mediate circRNA knockdown.^91^ The siRNA molecule is 21–23 nucleotides in length and has a highly specific structure that prevents silencing of erroneous genes.^96^ Typically, siRNA targets the circRNA-specific back-splicing junction structure to precisely knock down circRNA rather than its corresponding linear mRNA.^91^^,^^97^ shRNA is characterized by a ring structure and base-paired stem, which are processed into siRNA by Dicer enzyme and function in silencing target mRNA.^91^ Delivery of siRNA and shRNA in lipid-based polymers is currently the most convenient method for knockdown of circRNAs *in vivo*.^91^

Given the potential of RNAi therapy to treat a variety of cancers, scientists have applied RNAi strategies to RBP targets and demonstrated effectiveness in preclinical studies.^6^ For example, in studies of non-small cell lung cancer and ovarian cancer, *in vitro* targeted nanoparticle delivery of HUR-RNAi had a significant therapeutic effect in inhibiting cancer development.^98^^,^^99^ Notably, circRNA has a great advantage as a therapeutic strategy targeting RBP function in cancer because of itis stability and long half-life.^6^ Therefore, the use of RBP-isolated circRNAs as therapeutic molecules for the treatment of cancer that overexpress a specific RBP is a feasible approach.^6^ Although no clinical applications have been reported, they are expected in the near future.^6^

#### RNA editing

RNA editing is based on A-to-I editing by ADARs; inosine (I) is recognized by the cellular machinery as guanosine (G), making ADARs useful for A-to-G editing and associated protein sequence recoding.^90^^,^^100^ Therapeutic RNA editing uses antisense oligonucleotides to recruit endogenous ADARs, inducing ADARs to edit specific adenosines *in vitro* and *in vivo*.^101^ ADAR1 has been reported to inhibit circHIPK3 biogenesis because of A-to-I editing, and to mediate the expression of the multidrug resistance protein MRP4 in human kidney cells. The researchers stated that the level of RNA editing on pre-mRNA is underestimated because the abundance of circHIPK3 precursors is too high, and the lack of pre-mRNA makes RNA editing undetectable.^49^ To achieve programmable endogenous RNA regulation, the problem of exogenous ADAR delivery needs to be overcome. Antisense guide RNA (gRNA) for site-specific editing of mRNA is a good tool for RNA editing.^90^^,^^100^ The efficiency and specificity of gRNAs have become a top priority in the development of RNA editing therapies.^90^ However, RNA editing is a transient event that is often diluted as mRNA is converted.^90^ To improve RNA editing efficiency and extend the editing time, circular ADAR-recruiting guide RNA (cadRNA) was developed and tested to recruit endogenous ADARs.^90^ Compared with the use of linear gRNAs, the use of circular gRNAs in ADAR greatly prolongs the persistence of RNA editing *in vitro* and *in vivo*.^102^ Furthermore, correcting missense and nonsense mutations is the most logical application of RNA editing. Two recent studies have shown that RNA editing is a safer and more effective regimen for treating A1-antitrypsin deficiency caused by SERPINA1 (serpin family A member 1) missense mutations.^103^^,^^104^ With the clinical application of RNA editing technology, true precision and personalized medicine may be achieved.

#### CRISPR/Cas system

As a tool for large-scale genetic screening, the CRISPR-Cas system has been used to analyze gene function and biological pathways associated with human diseases, including cancers, driving another revolution in the field of biotechnology.^105^ The efficiency of CRISPR-Cas knockdown of circRNA is comparable to RNAi knockdown, but the off-target effect is greatly reduced, which is very suitable for systematic evaluation of circRNA function.^106^ However, targeting circRNAs using the CRISPR/Cas system is more challenging than directly targeting RNAi-based strategy after splicing.^91^ In general, CRISPR/Cas9 knocks out circRNA by disrupting intron pairing on both sides of the circular exon, which typically occurs during circRNA biogenesis.^107^ A previous study showed that knocking out circHIPK3 using CRISPR/Cas9 *in vitro* inhibited cell proliferation without affecting linear mRNA.^108^ Both CRISPR/Cas9 and CRISPR/Cas13 have the potential to knock out or knock down specific circRNAs and may have future clinical applications.^91^ CRISPR/Cas13 can directly target the back-splicing junction structure of circRNAs.^89^ However, because of the limited efficiency in delivering large-sized circRNAs, the delivery of synthetic circRNAs can be difficult.^91^ In addition, producing large amounts of circRNAs is not a realistic approach. Expanding the understanding of the functional mechanisms of circRNAs and developing specific and effective methods to target circRNAs *in vivo* will be key to advancing the clinical potential of circRNA-based therapies.^91^

In addition to the great advances the CRISPR system has made in targeting circRNA therapy, scientists have also used this molecular tool to further explore the role of RBP as a target for cancer therapy. Selective targeting of tumor cells and specific recognition of RBP molecules are necessary conditions for the CRISPR system to perform anti-tumor function.^109^ By high-throughput CRISPR screening *in vivo*, RBP HNRNPL (heterogeneous nuclear ribonucleoprotein L) was identified as a prostate cancer-dependent gene. It plays an important role in prostate cancer cell growth, targeting androgen receptors by regulating selective RNA splicing and circRNA formation.^110^ The m^6^A writing complex METTL3 was identified by CRISPR-Cas9 screening as the top candidate gene for lipopolysaccharide-activated macrophages.^111^ Recently, Wang et al used CRISPR-Cas9 screening technology to establish an up-regulated, physically interacting RBP network in acute myeloid leukemia, providing a strategy for treating acute myeloid leukemia that carries an RBP splicing mutation.^92^

Several studies have also attempted to use CRISPR-based therapies to treat cancer. Recent studies demonstrated that CRISPR-Cas9 gene-edited T cells are safe and feasible for clinical application. This conclusion was validated in two human phase I clinical trials involving patients with advanced non-small-cell lung cancer and children with refractory B-cell leukemia.^112^^,^^113^ While the CRISPR system has not yet been used to target RBPs for cancer therapy, the research results are encouraging and provide broad prospects for the application of targeting RBP in cancer therapy.^6^

## Conclusion

RBPs play a crucial role in regulating various biological activities.^114^ The interactions between circRNAs and RBPs modulate the production of circRNAs, thereby influencing the occurrence and progression of diseases. Researchers have explored the interaction mode of circRNA-RBP and revealed a large circRNA-RBP interaction network. In this review, we have discussed several representative RBPs that regulate circRNA production and function in tumors, including SP1, QKI, FUS, ADAR, and DHX9. We present an overview on the structural characteristics of RBPs and circRNAs and the molecular basis of their binding. We have discussed how RBPs bind to circRNAs to activate cancer pathways or transmit information that promotes tumor development. We also have reviewed the factors that influence circRNA-RBP interactions in the TME. Exploring the mechanisms of tumorigenesis and seeking effective methods of tumor treatment has been a hot spot in modern tumor biology research.^115^ Here we have discussed the current research on therapies targeting circRNA and RBP, focusing on RNA-based therapeutic approaches targeting the circRNA-RBP system. Research has identified many RBPs that are specifically expressed in various cancers and demonstrated to be key regulators of tumor development.^110^ The structural properties of circRNA and its resistance to RNA decay mechanisms make it an ideal diagnostic biomarker and therapeutic target.^116^ The development of traditional drugs takes time and effort to develop, and these treatments are frequently associated with many side effects.^117^ In contrast, the unique structural and biochemical properties of RNA allow it to be designed to a specific target.^118^ RNA therapy uses the principle of complementary base pairing, with RNA acting as a template, catalyst, scaffold, or modulator to target therapy in a programmable manner.^118^ The use of RNA therapy holds promise for previously difficult-to-treat human diseases, particularly cancer.^119^ Therefore, researchers are exploring how to combine the advantages of circRNAs with the function of RBPs to develop more effective RNA therapies.^120^

Although there have been numerous research reports, challenges remain in the research of circRNA-RBP network because of theoretical deficiencies and technical limitations. For example, the identification of RBPs involved in circRNA function and the mechanisms by which RBPs regulate circRNA production have not been full determined. Additionally, more databases are needed to determine the precise structure of circRNAs outside the binding site to explain the conformational and functional changes of proteins. Finally, targeting circRNAs and RBPs represents the frontier of RNA therapy, but clinical application requires further research addressing specificity, delivery methods, and immunogenicity. For example, the off-target effect of RNA from the imprecise binding of therapeutic RNA molecules to a target may cause a variety of side effects.^121^ The choice of delivery method is related to the effectiveness of RNA therapy.^122^ The immunogenicity problem is the immune tolerance caused by the pathogen molecules.^115^ The ultimate success of RNA therapy requires the application of multiple interdisciplinary approaches, including technological advances in molecular biology, immunology, pharmacology, chemistry, and nanotechnology.^123^ Researchers from different disciplines have improved RNA therapy techniques in various ways to reduce off-target effects. Many new molecular materials and delivery methods have been developed, such as nanomaterials and viral vectors.^124^ Better understanding of circRNA-RBP interactions through additional research may lead to the development of RNA therapies, bringing hope to cancer patients.

## Funding

This work was supported by the National Science Foundation of Guangdong Province, China (No. 2022A1515011731, 2021A1515011062), the Guangdong Provincial Administration of Traditional Chinese Medicine (China) (No. 20221211), the Project of Zhanjiang City, Guangdong, China (No. 2020A01016, 2020B01346, 2021A05077, 2016B01062), and the Affiliated Hospital of Guangdong Medical University (China) (No. 4SG21231G, LCYJ2017A003, CLP202113001, CLP2021B001, LCYJ2020B008, BK201616).

## CRediT authorship contribution statement

**Lixia Li:** Conceptualization, Methodology, Writing – original draft. **Chunhui Wei:** Writing – original draft. **Yu Xie:** Writing – review & editing. **Yanyu Su:** Writing – review & editing. **Caixia Liu:** Writing – review & editing. **Guiqiang Qiu:** Writing – review & editing. **Weiliang Liu:** Writing – review & editing. **Yanmei Liang:** Writing – review & editing. **Xuanna Zhao:** Supervision. **Dan Huang:** Resources. **Dong Wu:** Conceptualization, Project administration.

## Conflict of interests

The authors declared no competing interests.
